# Variation in nutritional quality of *Vicia sativa* across climatic gradients and development of a comprehensive forage quality index

**DOI:** 10.1371/journal.pone.0356688

**Published:** 2026-08-28

**Authors:** Wei Xu, Zhuxin Mao

**Affiliations:** 1 School of Biological and Environmental Engineering, Xi’an University, Xi’an, China; 2 Xi’an Botanical Garden of Shaanxi Province (Institute of Botany of Shaanxi Province), Xi’an, Shaanxi, China; University of Minnesota, UNITED STATES OF AMERICA

## Abstract

*Vicia sativa* is a leguminous forage crop with important ecological and feeding value, and its nutritional quality is closely influenced by its growing environment. Field experiments were conducted over one growing season across three ecological zones: the cold, humid alpine region of the Qinghai-Tibet Plateau (XH), the humid transitional zone between the Qinghai-Tibet and Loess Plateaus (MX), and the semi-arid Loess Plateau (QY). Four *V. sativa* cultivars were evaluated using a randomized complete block design with three replicates. Nutritional components, including soluble sugars, crude protein, crude fat, and multiple fatty acids, were measured. Correlation analysis and principal component analysis (PCA) were performed to evaluate relationships among nutritional traits. A comprehensive forage nutritional quality index (CFNQI) was developed to assess forage quality quantitatively. Results indicated that both ecological zone and cultivar significantly affected nutritional traits, although ecological effects were generally stronger than cultivar effects for most traits. XH and MX samples showed higher CFNQI values than those from QY.. Specifically, *V. sativa* grown in XH was characterized by higher soluble sugar and unsaturated fatty acid contents, whereas samples from MX showed relatively greater crude fat and unsaturated fatty acid accumulation. In contrast, forage quality in QY was limited due to warm, dry conditions. This study highlights ecological differences in nutritional traits of *V. sativa* and supports the optimization of regionally adapted forage cultivation and high-quality forage resources.

## 1. Introduction

Grassland ecosystems cover approximately 40.5% of the Earth's land surface [1,2], and play critical roles in ecological regulation, livestock production, grasslands contribute significantly to water conservation, carbon sequestration, and the maintenance of biodiversity [3–5]. However, climate change and intensified human activities have led to widespread grassland degradation, manifested in reduced vegetation cover, declining forage quality, and diminished productivity [6–9]. Consequently, restoring grassland ecological functions and enhancing forage nutritional value have become urgent priorities in grassland management and research.

Leguminous forage species are widely utilized in grassland establishment, ecological restoration, and livestock production due to their strong nitrogen-fixing capacity, rapid growth, and high palatability [10,11]. *Vicia sativa*, a widely adaptable leguminous forage, is particularly suitable for cultivation in the plateau, hilly, and arid to semi-arid regions [12–14]. In Northwest China, where grassland ecosystems are often constrained by low soil fertility, water scarcity, and climatic variability, *V. sativa* plays an important ecological role. Its strong nitrogen-fixing capacity enhances soil nitrogen availability and improves soil fertility, which is critical for the restoration of degraded grasslands. In addition, its adaptability to cold, drought-prone, and nutrient-poor environments makes it a valuable species for stabilizing vegetation and supporting sustainable livestock production [15,16]. However, current research on the mechanisms underlying forage quality formation and ecological adaptability of *V. sativa* across diverse ecological zones in Northwest China remains limited. This knowledge gap hampers its large-scale promotion and refined management under the region’s complex climatic conditions.

The growth, development, and nutrient accumulation of *V. sativa* are regulated by a range of environmental factors, among which climatic conditions act as primary drivers [17,18]. Variations in temperature, precipitation, and solar radiation across distinct climatic zones result in pronounced differences in plant carbon-nitrogen metabolism, photosynthetic efficiency, and fatty acid biosynthesis, ultimately shaping forage nutritional quality. These environmental factors regulate nutrient accumulation through their effects on key physiological processes. Temperature and light intensity influence photosynthetic efficiency and carbon assimilation, thereby affecting soluble sugar accumulation. Water availability regulates stomatal conductance and nutrient transport, influencing both carbon and nitrogen metabolism. In addition, temperature-dependent enzymatic activities play an important role in fatty acid biosynthesis, particularly in the desaturation process, which determines the proportion of unsaturated fatty acids. For instance, regions characterized by high elevations and large diurnal temperature fluctuations often exhibit intense solar radiation, which favors the accumulation of soluble sugars and unsaturated fatty acids [19,20]. In contrast, temperate and humid climates tend to promote protein synthesis and optimize the nutritional profile of forage [21,22]. In arid and hot regions, however, water stress commonly leads to suppressed carbon and nitrogen metabolic efficiency, thereby reducing overall forage quality [23]. Against the backdrop of Northwest China's complex topography and climatic gradients, elucidating the ecological patterns and regulatory mechanisms underlying nutrient composition in *V. sativa* across different environmental types is of fundamental importance. Such insights are essential for the ecologically informed selection of forage species and the strategic modulation of forage quality traits in variable environments.

Forage nutritional quality represents a fundamental determinant of its feeding value. While conventional parameters, such as crude protein, soluble sugars, and crude fiber, remain essential indicators, they are often insufficient to capture the full spectrum of nutrient requirements for livestock and ruminants [24,25]. Increasing evidence highlights the pivotal role of fatty acid composition, particularly the proportion of unsaturated fatty acids, in assessing the potential of forage to promote animal health, modulate milk fat profiles, and enhance meat quality [26–28]. Given the complexity of environmental factors on forage quality, reliance on a single nutritional component is inadequate for comprehensive evaluation. Therefore, establishing an evaluation system and quantitative indicators that integrate multiple key nutritional factors is essential for the precise assessment of forage quality.

In this study, three representative climatic zones in Northwest China were selected: the cold and humid climatic zone of the Qinghai-Tibet Plateau, the humid transitional zone from the Qinghai-Tibet Plateau to the Loess Plateau, and the temperate semi-arid climatic zone of the Loess Plateau. A growth adaptability experiment of *V. sativa* was conducted across these zones. The objectives of this study were to: (1) evaluate the variation in nutritional composition of *V. sativa* across different ecological gradients; (2) analyze the fatty acid composition and its ecological responses; and (3) develop a comprehensive forage nutritional quality index (CFNQI) to assess regional differences in forage quality.

## 2. Materials and methods

### 2.1. Experimental sites

**Fig 1:**
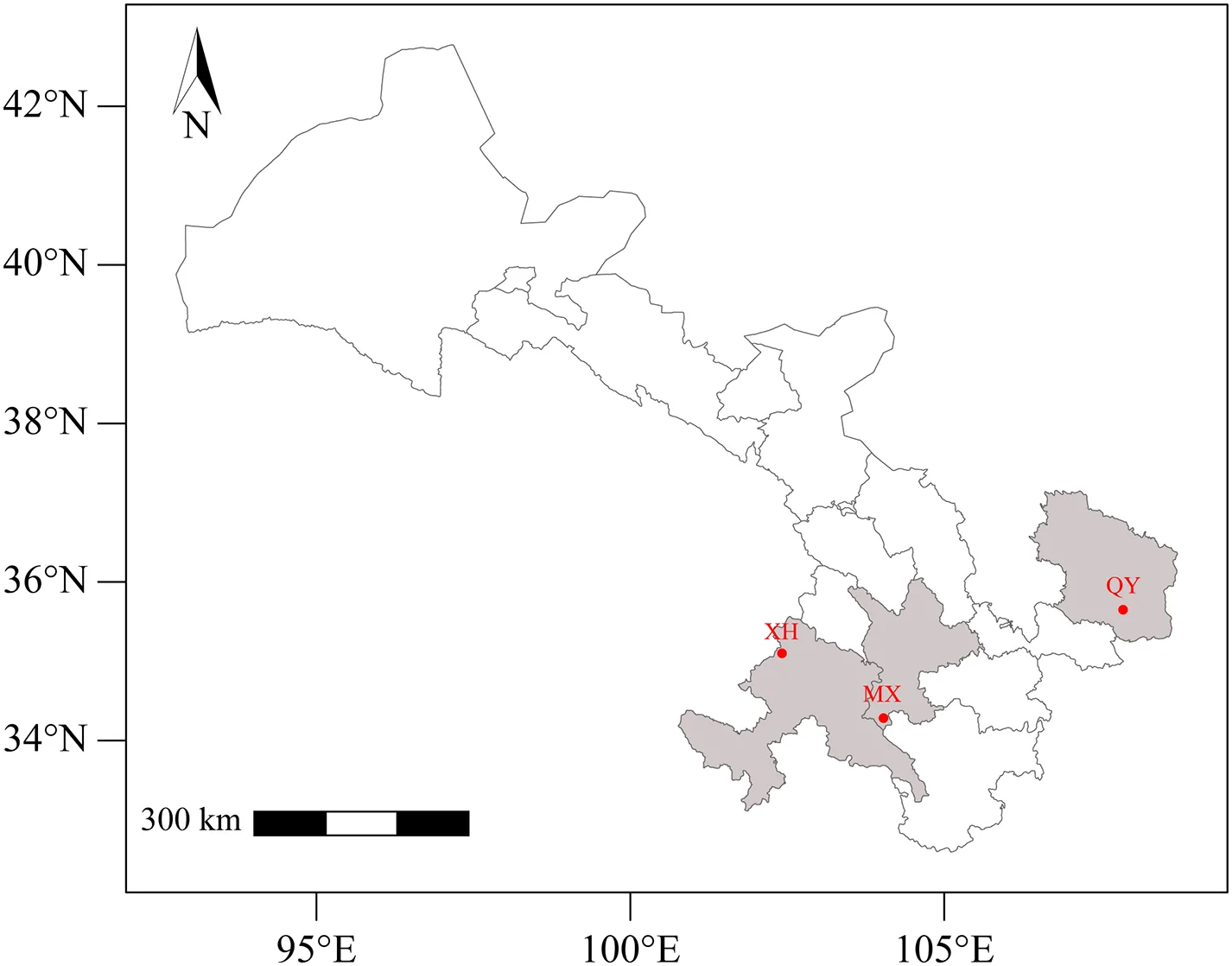
Geographic distribution of the experimental sites across the ecological gradient. Contains information from OpenStreetMap and OpenStreetMap Foundation, which is made available under the Open Database License.

This study was conducted at three sites located in distinct climatic zones of Gansu Province, China: Xiahe County (XH), Minxian County (MX), and Qingyang County (QY). XH (102°25′E, 35°06′N, elevation: 3050 m) is situated in the cold and humid climatic zone of the Qinghai-Tibet Plateau, characterized by low mean annual temperatures and relatively high precipitation during the growing season. The dominant soil type at this site is chernozem. MX (104°02′E, 34°17′N, elevation: 2535 m) lies within the humid transitional zone between the Qinghai-Tibet Plateau and the Loess Plateau, with moderate temperature and precipitation conditions. Its soil type is alpine meadow soil. QY (107°51′E, 35°39′N; elevation: 1318 m) is located in the temperate semi-arid climatic zone of the Loess Plateau, characterized by higher temperatures and lower precipitation. The predominant soil type is calcic cinnamon soil. No specific permits were required for the described field studies, and the study did not involve endangered or protected species. Key climatic characteristics of the experimental sites are summarized in Table 1. These data were obtained from nearby meteorological stations. The geographical distribution of the sampling sites was shown in Fig 1. The map background was created using data from OpenStreetMap (OpenStreetMap contributors, 2024). Contains information from OpenStreetMap and OpenStreetMap Foundation, which is made available under the Open Database License.

**Table 1:** Key climatic characteristics of the experimental sites.

Parameter	XH	MX	QY
Mean annual temperature (°C)	3.1	5.2	9.1
Annual precipitation (mm)	510–590	700–750	480–660
Frost-free period (days)	56–70	90–120	140–180
Sunshine duration (h)	2296	2214	2800

### 2.2. Experimental materials

Field experiments were conducted at the three study sites: XH, MX, and QY. The experimental materials consisted of three *V. sativa* lines differing in maturity: an early-maturing line (2505), a mid-maturing line (2560), and a late-maturing line (2556), along with a standard cultivar used as control (333/A). Each plot measured 4 m × 4 m, and a randomized complete block design was employed with three replicates. Each replicate consisted of one block, within which all four cultivars were randomly assigned to plots to minimize the effects of environmental heterogeneity. Plots within each block were arranged with adequate spacing to reduce border effects. The seeding rate was 75 kg/hm^2^, using row seeding at a depth of 2–3 cm. Basal fertilizer was incorporated into the soil before sowing, and topdressing was applied during the branching stage by furrow placement at a depth of 5 cm. Before sowing at each site, soil nutrient levels in the topsoil layer (0–30 cm) were measured. The following dates were April 25 for the XH site, April 13 for MX, and April 2 for QY. During the experimental period, pest and disease control was carried out promptly as needed, and weeds were removed promptly. Harvesting was conducted at the early flowering stage (20% of plants flowering), with a cutting height of approximately 3 cm. Plants from the central area of each plot were harvested to avoid border effects. All harvested biomass within the sampling area was weighed immediately after cutting. A representative subsample (~1000 g) was then randomly collected from the mixed fresh forage of each plot, thoroughly homogenized, and used for subsequent analyses. Fresh forage was inactivated at 105 °C for 15 min, and then oven-dried at 65°C for 24 h. The dry weight was recorded to calculate hay yield. Dried samples were ground and passed through a 0.2 mm sieve, and the resulting powders were stored in brown glass bottles for subsequent nutritional analyses.

### 2.3. Meteorological data acquisition

The meteorological data used in this study for the year 2023 were primarily obtained from publicly available sources provided by the China Meteorological Administration and affiliated meteorological stations. The specific parameters include annual mean temperature, monthly mean temperature, annual precipitation, monthly precipitation, frost-free period, and annual sunshine duration for each experimental site. The main data sources were the National Meteorological Science Data Center (https://data.cma.cn/) and local meteorological bureaus. The meteorological data collection period covered the entire experimental duration and was adjusted according to the specific conditions of each site to ensure data representativeness and reliability.

### 2.4. Soil fertility measurement

Soil samples were collected from the topsoil layer (0–30 cm) at each experimental site before sowing. At each site, soil was sampled from multiple points within each plot and combined to form a composite sample. Three independent composite samples (replicates) were collected per site and used for subsequent analyses. Soil total organic matter and total nitrogen content were measured using a carbon-nitrogen analyzer (Vario Max CN, Elementar, Germany). Total phosphorus content was determined by the ammonium molybdate spectrophotometric method, while available potassium content was analyzed by flame atomic absorption spectrometry [29]. Soil pH was measured in a soil-to-water suspension (ratio 1.0:2.5) using a pH meter (Sartorius PB-10, Göttingen, Germany) [29].

### 2.5. Determination of nutritional components and fatty acid content

Soluble sugar content was determined using the anthrone–sulfuric acid methods, following previously described procedures [30]. Crude protein content was measured using the micro-Kjeldahl method [31]. Crude fat content was determined according to standard AOAC method [32]. For fatty acid analysis, lipids were first extracted from dried samples using an organic solvent extraction method (chloroform–methanol system). The extracted lipids were then converted to fatty acid methyl esters (FAMEs) prior to analysis. The composition and content of fatty acids were analyzed using gas chromatography-mass spectrometry (GC-MS). Fatty acid composition was determined using gas chromatography–mass spectrometry (GC–MS) [16]. Quantification of fatty acids was performed using external standards, and calibration curves were established using known concentrations of standard fatty acid mixtures. Instrument parameters, including column type, carrier gas flow rate, and temperature program, were set according to standard protocols to ensure analytical accuracy and reproducibility.

### 2.6. Evaluation of forage nutritional quality

A Comprehensive Forage Nutritional Quality Index (CFNQI) was developed to evaluate the overall nutritive value of *V. sativa*. The CFNQI was developed as a species-specific evaluation index for *V. sativa* under the ecological conditions and forage utilization requirements of Northwest China. The index was designed to integrate major nutritional traits relevant to its feeding value in regional livestock production systems. The weighting coefficients were determined based on the relative importance of each trait in *V. sativa* forage quality evaluation, supported by published studies and expert knowledge on legume forage utilization in Northwest China [33–36]. Crude protein and soluble sugar are the primary determinants of forage nutritive value, influencing palatability, energy, and nitrogen supply. Crude fat reflects the energy density of the forage. A higher proportion of total unsaturated fatty acids generally indicates better forage quality, whereas excessive total saturated fatty acids are associated with less favorable nutritive characteristics. The index is calculated as follows:


$$\begin{array}{c} \text{CFNQI}=\;\text{0}.\text{3}\text{5}\cdot SS^{\prime }+\;\text{0}.\text{3}\text{5}\cdot CP^{\prime }+\;\text{0}.\text{1}\text{5}\cdot CF+\text{0}.\text{2}\cdot TUFA^{\prime }-\text{0}.\text{1}\cdot TSFA^{\prime } \end{array}$$ (1)

Where $SS^{\prime }$ denotes the standardized soluble sugar content, $CP^{\prime }$ the standardized crude protein content, CF the standardized crude fat content, $TUFA^{\prime }$ the standardized total unsaturated fatty acids, and $TSFA^{\prime }$ the standardized total saturated fatty acids.

### 2.7. Statistical analysis

Experimental data were organized using Microsoft Excel 2019. Statistical analyses were performed using R software. One-way ANOVA followed by Duncan’s multiple range test was used to assess differences among treatments. Correlation analysis and principal component analysis (PCA) were conducted to evaluate relationships among variables. Graphical visualization was performed using the ggplot2 package.

## 3. Results

### 3.1. Climate and soil characteristics of experimental sites

**Fig 2:**
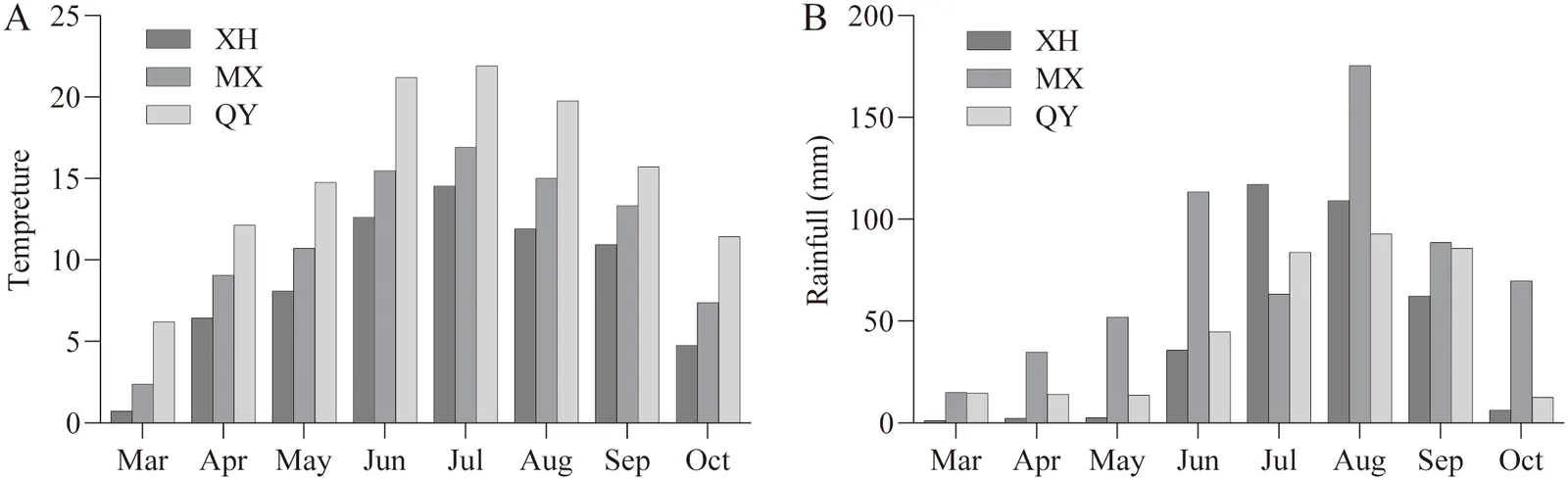
Temperature and precipitation conditions in the experimental regions. (A) Monthly average air temperature in each experimental site. (B) Monthly average precipitation in each experimental site.

From March to October, the three experimental sites exhibited distinct climatic differences. The XH site showed the lowest monthly mean temperatures among the three sites, rising from 0.71 °C in March to 14.52 °C in July. It also received the least precipitation, with monthly rainfall ranging from 1.13 mm to 117.02 mm, primarily concentrated between June and August. The MX site had intermediate temperatures, increasing from 2.38 °C in March to 16.90 °C in July, and relatively higher precipitation, peaking at 175.4 mm in August. The QY site exhibited the highest temperatures, from 6.19 °C in March to 21.90 °C in July, with abundant summer rainfall reaching 92.8 mm in August (Figs 2A, 2B).

Regarding soil baseline fertility, the XH site exhibited the highest levels of soil organic matter (42.5 g/kg) and nitrogen content (4.55 g/kg), indicating relatively strong soil fertility. The MX site showed moderate nutrient levels, with notably higher phosphorus content (1.25 g/kg). The QY site had the lowest organic matter and nitrogen contents but the highest potassium content (22.8 g/kg). Soil pH values differed among the three sites, with XH exhibiting neutral conditions (pH 7.18), while MX and QY were slightly alkaline, reflecting distinct regional soil physicochemical characteristics (Table 2).

**Table 2:** Baseline soil fertility characteristics of experimental sites.

Zone	Organic matter (g/kg)	N (g/kg)	P (g/kg)	K (g/kg)	pH value
XH	42.50 ± 1.14a	4.55 ± 0.20a	0.71 ± 0.07b	21.60 ± 0.80a	7.18 ± 0.13b
MX	22.90 ± 1.85b	1.47 ± 0.13b	1.25 ± 0.10a	18.70 ± 0.71b	8.43 ± 0.13a
QY	11.20 ± 0.98c	0.78 ± 0.08c	0.65 ± 0.05b	22.80 ± 0.79a	8.67 ± 0.12a

Note: Data are expressed as mean ± SD (n = 3). Different lowercase letters within the same column indicate significant differences among cultivars within the same ecological zone (*p* < 0.05). Different uppercase letters within the same column indicate significant differences among ecological zones for the same cultivar (*p* < 0.05), according to one-way ANOVA followed by Duncan’s multiple range test.

### 3.2 Accumulation characteristics of dry matter components in V. sativa

Significant differences in soluble sugar, crude protein, and crude fat contents of *V. sativa* were observed among the three planting regions (Table 3). In the XH site, all accessions exhibited relatively high soluble sugar content, with the highest value recorded in accession 2505 (9.39%) and an overall average of 7.90%, exceeding that of the MX site (7.32%) and QY site (6.15%). This indicates that the cold, high-altitude environment in the XH site is conducive to carbohydrate accumulation. Crude protein content showed little variation among the three sites, maintaining a relatively stable level of around 26%, suggesting that nitrogen metabolism in *V. sativa* is less sensitive to the ecological differences among regions. In contrast, crude fat content was significantly higher in the MX site, with an average of 1.63% and a maximum of 1.75% in accession 2505. The lowest fat content was observed in QY, with an average of only 1.12%. These results indicate that ecological factors, particularly climatic and soil conditions, have a marked impact on the accumulation of dry matter components in *V. sativa* (Table 3). Low temperatures favor the accumulation of soluble sugars, while moderate temperature and adequate precipitation are more favorable for lipid synthesis and storage. Compared with the climatic zone factor, the cultivar factor had a smaller effect on dry matter content.

**Table 3:** Dry matter composition of *V. sativa* across different ecological zones.

Zone	Cultivars	Soluble sugar (%)	Crude protein (%)	Crude fat (%)
XH	2505	9.39 ± 0.13aA	26.98 ± 0.18aA	1.39 ± 0.06aB
	2556	7.19 ± 0.12cA	26.58 ± 0.18bAB	1.37 ± 0.06aB
	2560	7.53 ± 0.13bA	26.28 ± 0.17bA	1.11 ± 0.07bB
	333/A	7.48 ± 0.13bA	26.55 ± 0.17bC	1.36 ± 0.06aB
MX	2505	8.71 ± 0.11aB	26.15 ± 0.15bB	1.75 ± 0.07aA
	2556	5.63 ± 0.13dB	26.50 ± 0.20bB	1.54 ± 0.06bA
	2560	7.66 ± 0.12bA	26.13 ± 0.23bA	1.63 ± 0.05bA
	334/A	7.29 ± 0.14cA	26.88 ± 0.18aA	1.58 ± 0.06bA
QY	2505	7.21 ± 0.12aC	23.86 ± 0.15cC	1.22 ± 0.06aC
	2556	4.80 ± 0.10cC	26.17 ± 0.13aA	0.98 ± 0.05bC
	2560	6.24 ± 0.14bB	26.03 ± 0.16aA	1.04 ± 0.04bA
	335/A	6.34 ± 0.15bB	25.63 ± 0.15bB	1.25 ± 0.05aB

Note: Data are expressed as mean ± SD (n = 3). Within each ecological zone, different lowercase letters indicate significant differences among cultivars (*p* < 0.05). Within each cultivar, different uppercase letters indicate significant differences among ecological zones (*p* < 0.05). Differences were analyzed using one-way ANOVA followed by Duncan’s multiple range test.

### 3.3. Effects of ecological zones and genotypes on dry matter accumulation

**Fig 3:**
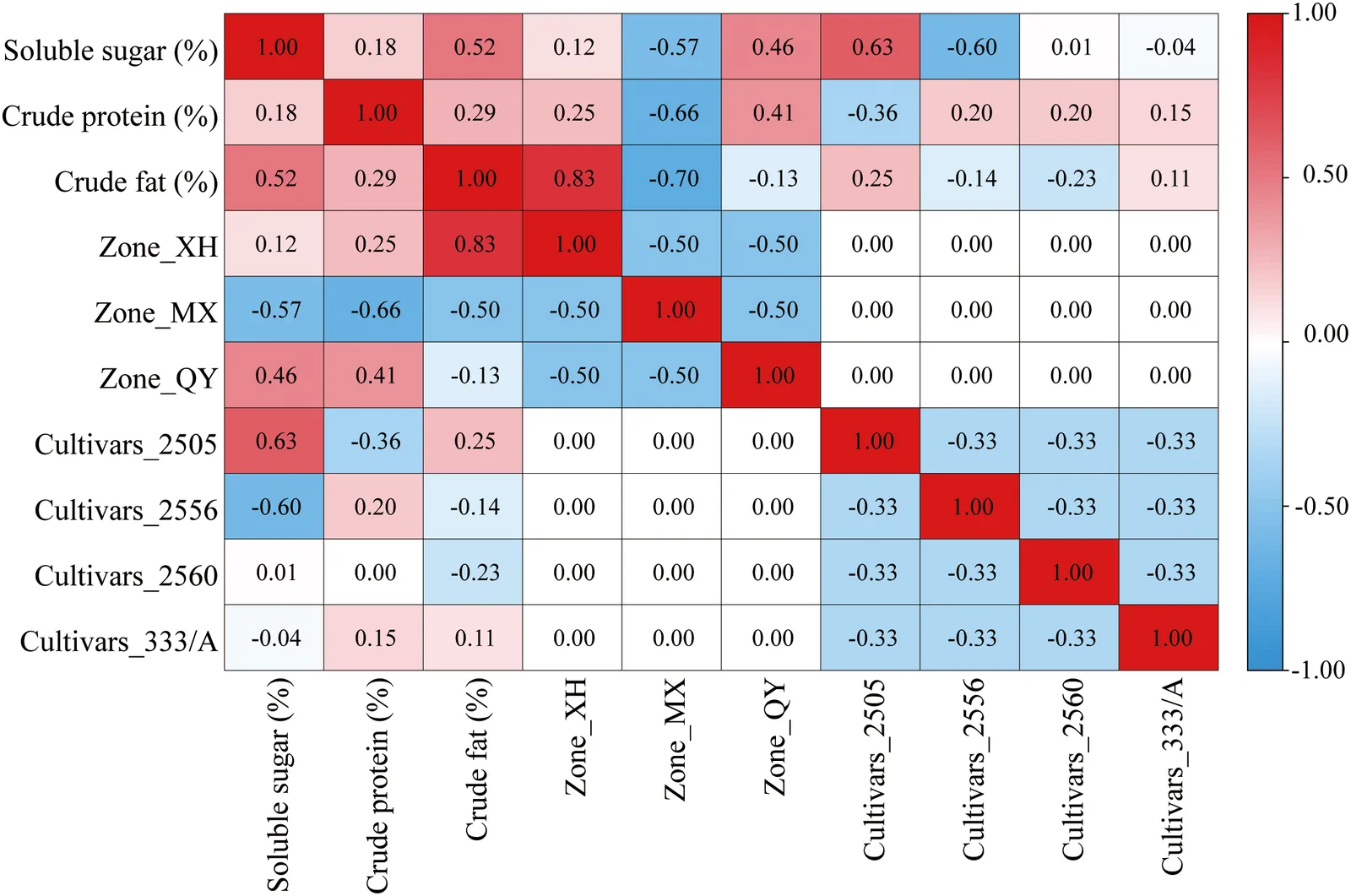
Correlation analysis between environmental and genetic factors and dry matter accumulation in *V. sativa.* The heatmap shows Pearson correlation coefficients among major saturated and unsaturated fatty acids. Positive correlations are shown in red and negative correlations in blue, with intensity indicating the strength of the correlation.

Correlation analysis revealed that there were notable associations among the three dry matter components of *V. sativa*. A moderate positive correlation (r = 0.52) was observed between soluble sugar and crude fat content, indicating that samples with higher sugar levels tend to accumulate more fat. In contrast, the correlation between soluble sugar and crude protein was relatively weak (Fig 3). Regarding environmental effects, the XH site exhibited a strong positive correlation with crude fat content (r = 0.83). The QY site showed moderate positive correlations with soluble sugar and crude protein contents, with correlation coefficients of 0.46 and 0.41, respectively (Fig 3). Ecological factors play a particularly prominent role in determining fat content, while genotype has a pronounced effect on soluble sugar accumulation.

### 3.4 Accumulation characteristics of fatty acid components in V. sativa

Significant differences were observed in the composition and content of major fatty acids in *V. sativa* across different ecological zones and genotypes. Among the three regions, plants grown in the MX site exhibited the highest total fatty acid (TFA) content and total unsaturated fatty acid (TUFA) levels. Notably, variety 2505 in this region accumulated up to 10.00 g/kg of TUFA, with the highest recorded TFA content reaching 13.28 g/kg. The ratio of unsaturated to saturated fatty acids (UFA/SFA) reached 3.05, indicating superior fatty acid quality. In contrast, the QY site presented lower fatty acid accumulation. For instance, variety 2560 in this zone had a TUFA content of only 2.92 g/kg and the lowest TFA content at 5.06 g/kg. The UFA/SFA ratio was markedly lower, suggesting a higher proportion of saturated fatty acids and, consequently, a lower nutritional quality of the fatty acid profile. The XH site exhibited intermediate levels of fatty acid accumulation. Among all tested genotypes, variety 2505 demonstrated the highest overall TFA content and the most favorable unsaturated-to-saturated fatty acid ratio across multiple environments (Table 4). These results suggest that the environmental conditions in the MX zone are particularly conducive to the biosynthesis and accumulation of unsaturated fatty acids. Furthermore, variety 2505 exhibits stable and superior fatty acid quality, making it a promising candidate for targeted cultivation in high-nutritional-value forage production systems.

**Table 4:** Fatty acid composition of *V. sativa* in different ecological zones and cultivars (g/kg DM).

Zone	Cultivar	SFA			
		Palmitic_Acid	Stearic_Acid	Arachidic_Acid	Behenic_Acid
XH	2505	2.16 ± 0.13aA	0.38 ± 0.02aA	0.24 ± 0.02aB	0.18 ± 0.01aC
	2556	1.56 ± 0.10cB	0.14 ± 0.01cC	0.24 ± 0.02aB	0.18 ± 0.01aB
	2560	1.44 ± 0.08cB	0.30 ± 0.02bB	0.24 ± 0.02aB	0.16 ± 0.01aC
	333/A	1.86 ± 0.10bB	0.38 ± 0.03aB	0.26 ± 0.02aB	0.18 ± 0.01aC
MX	2505	2.36 ± 0.14aA	0.42 ± 0.03aA	0.30 ± 0.02aA	0.20 ± 0.01bB
	2556	2.06 ± 0.10bA	0.16 ± 0.01bA	0.30 ± 0.02aA	0.22 ± 0.02abA
	2560	2.32 ± 0.12aA	0.42 ± 0.03aA	0.32 ± 0.03aA	0.24 ± 0.02aA
	333/A	2.18 ± 0.12abA	0.44 ± 0.03aA	0.28 ± 0.02aAB	0.20 ± 0.01bB
QY	2505	1.56 ± 0.10abB	0.30 ± 0.02aB	0.28 ± 0.02abA	0.22 ± 0.01aA
	2556	1.70 ± 0.09aB	0.08 ± 0.01cB	0.32 ± 0.03aA	0.24 ± 0.02aA
	2560	1.42 ± 0.07bB	0.26 ± 0.02bB	0.26 ± 0.02bB	0.20 ± 0.01aB
	333/A	1.66 ± 0.08aB	0.32 ± 0.03aC	0.32 ± 0.03aA	0.22 ± 0.01aA
Zone	Cultivar	UFA			
		Palmitoleic_Acid	Oleic_Acid	Linoleic_Acid	Linolenic_Acid
XH	2505	0.16 ± 0.01aB	0.36 ± 0.03bB	2.10 ± 0.22aB	5.64 ± 0.50aB
	2556	0.14 ± 0.01bA	0.26 ± 0.02cB	1.72 ± 0.15bB	4.88 ± 0.45aA
	2560	0.10 ± 0.01cB	0.30 ± 0.03cB	1.26 ± 0.10cB	4.00 ± 0.36bB
	333/A	0.10 ± 0.01cB	0.50 ± 0.04aA	1.86 ± 0.15abB	5.46 ± 0.50aA
MX	2505	0.20 ± 0.02aA	0.48 ± 0.04aA	2.58 ± 0.25aA	6.74 ± 0.70aA
	2556	0.16 ± 0.02bA	0.42 ± 0.03aA	2.04 ± 0.20bA	5.20 ± 0.50bA
	2560	0.16 ± 0.01bA	0.42 ± 0.03aA	2.36 ± 0.20abA	6.78 ± 0.70aA
	333/A	0.22 ± 0.02aA	0.49 ± 0.04aA	2.24 ± 0.22abA	5.76 ± 0.60abA
QY	2505	0.06 ± 0.01aC	0.30 ± 0.03aB	0.94 ± 0.10bcC	1.68 ± 0.20aC
	2556	0.08 ± 0.01aB	0.28 ± 0.03aB	1.22 ± 0.10aC	2.02 ± 0.22aB
	2560	0.08 ± 0.01aB	0.26 ± 0.02aB	0.86 ± 0.08cC	1.72 ± 0.18aC
	333/A	0.08 ± 0.01aB	0.26 ± 0.02aB	1.06 ± 0.12abC	1.82 ± 0.20aB
Zone	Cultivar	TSFA	TUFA	TFA	UFA/SFA
XH	2505	2.96	8.26	11.22	2.79
	2556	2.12	7.00	9.12	3.30
	2560	2.14	5.66	7.80	2.64
	333/A	2.68	7.92	10.60	2.96
MX	2505	3.28	10.00	13.28	3.05
	2556	2.74	7.82	10.56	2.85
	2560	3.30	9.72	13.02	2.95
	333/A	3.10	8.71	11.81	2.81
QY	2505	2.36	2.98	5.34	1.26
	2556	2.34	3.60	5.94	1.54
	2560	2.14	2.92	5.06	1.36
	333/A	2.52	3.22	5.74	1.28

Note: Data are expressed as mean ± SD (n = 3). Within each ecological zone, different lowercase letters indicate significant differences among cultivars (p < 0.05). Within each cultivar, different uppercase letters indicate significant differences among ecological zones (p < 0.05). Differences were analyzed using one-way ANOVA followed by Duncan’s multiple range test.

### 3.5. Correlation analysis among fatty acid components in V. sativa

**Fig 4:**
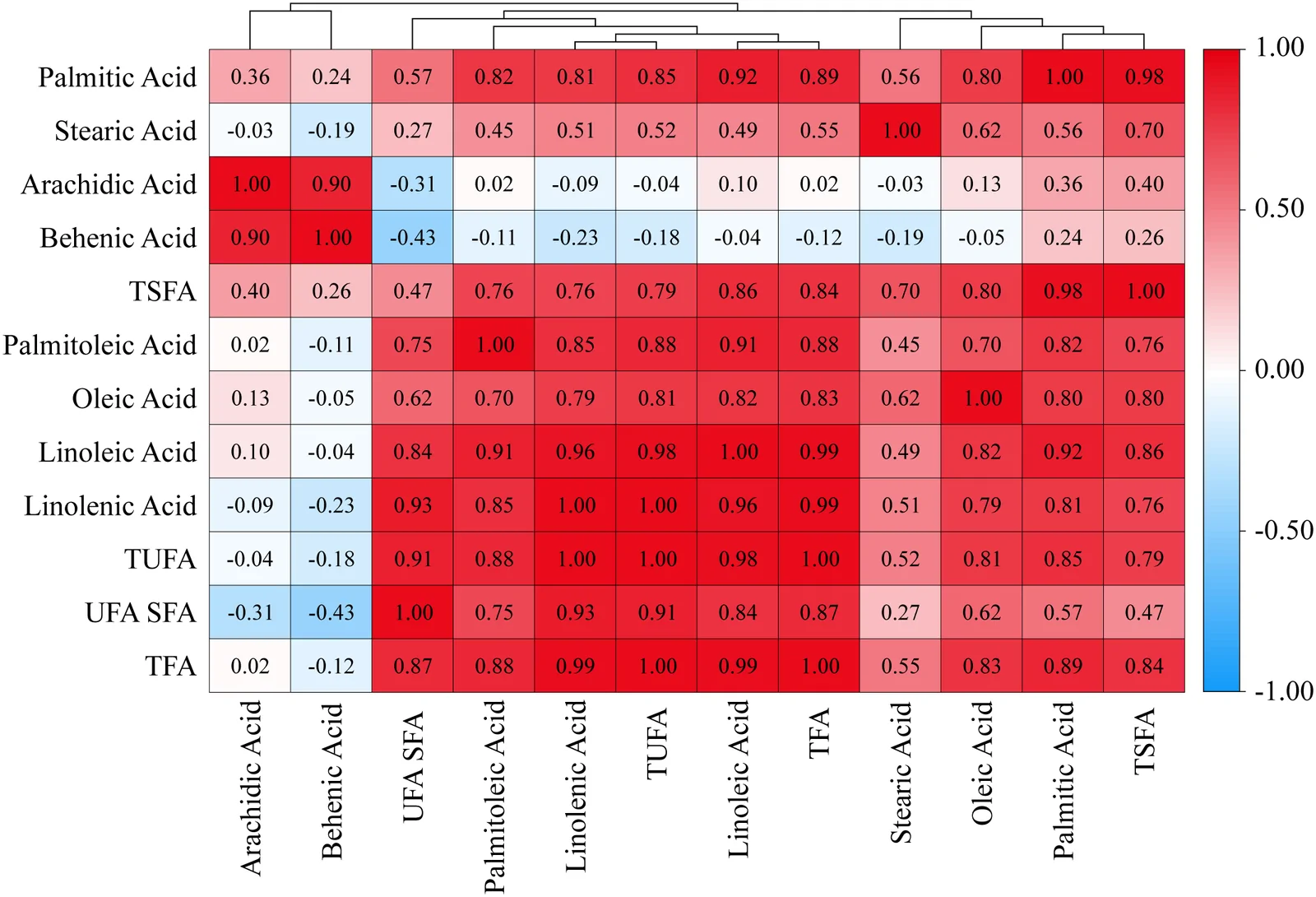
Correlation analysis among fatty acid components in *V. sativa.* The heatmap shows Pearson correlation coefficients among major saturated and unsaturated fatty acids. Positive correlations are shown in red and negative correlations in blue, with intensity indicating the strength of the correlation.

Within the saturated fatty acid (SFA) group, palmitic acid (C16:0) showed a moderate positive correlation with stearic acid (r = 0.56). Arachidic acid exhibited an extremely strong positive correlation with behenic acid (r = 0.90) (Fig 4). For unsaturated fatty acids (UFA), linoleic acid and linolenic acid showed an exceptionally strong positive correlation (r = 0.96). This indicates that these two polyunsaturated fatty acids are the major contributors to overall UFA accumulation in *V. sativa* (Fig 4). Additionally, palmitoleic acid also showed a strong correlation with linoleic acid (r = 0.91). Moreover, the UFA/SFA ratio was significantly positively correlated with both linoleic acid and linolenic acid (Fig 4).

### 3.6. Principal component analysis of fatty acid components

**Fig 5:**
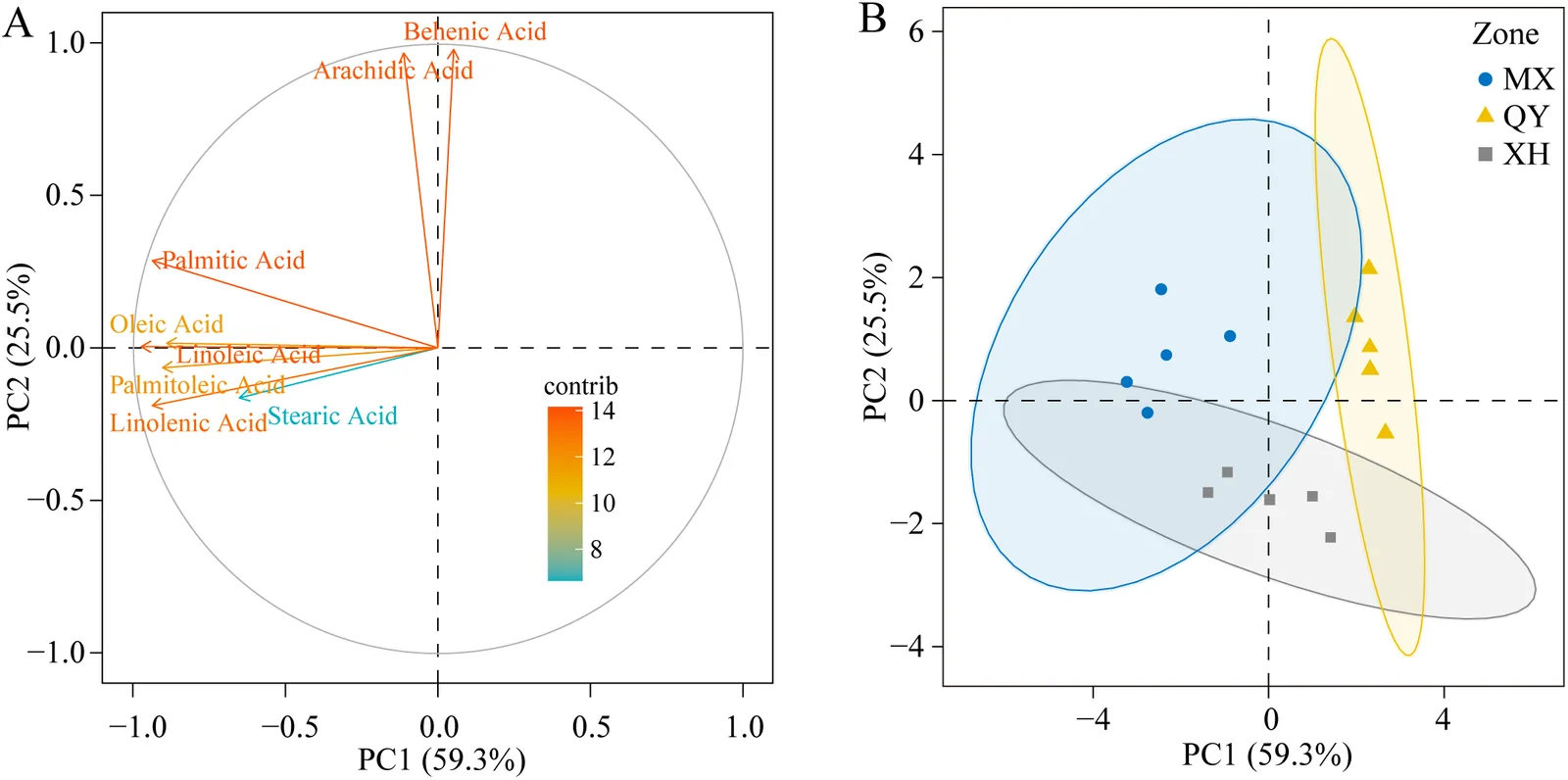
Principal component analysis (PCA) of fatty acid components in *V. sativa.* (A) Factor loading plot of the principal components. The major contributing fatty acids for PC1 and PC2 are indicated, showing their influence on sample differentiation. (B) Score plot of sample distribution based on PCA. Samples from different ecological regions were separated according to their fatty acid profiles, reflecting environmental and genetic effects.

To further investigate the variations in the fatty acid composition of *V. sativa* across different cultivars and ecological regions, a principal component analysis (PCA) was conducted on the major fatty acid components. The results indicated that the first two principal components collectively explained 84.8% of the total variance, effectively capturing the majority of information from the original dataset (Table 5). Among them, PC1 alone accounted for 59.3% of the variance and showed strong positive loadings with linoleic acid, linolenic acid, and oleic acid, reflecting the principal variation in unsaturated fatty acid content. PC2 explained 25.5% of the variance and was strongly negatively correlated with arachidic acid and behenic acid, highlighting the differences in long-chain saturated fatty acids (Fig 5A). The fatty acid composition of *V. sativa* displayed distinct ecological clustering patterns. Samples from the MX site were primarily distributed in the negative range of PC1, with a relatively scattered distribution along PC2, indicating moderate to high levels of unsaturated fatty acids with greater variability in long-chain saturated fatty acid content. Samples from the QY site were mainly located in the positive range of PC1 and clustered tightly along PC2, suggesting a consistent but lower accumulation of unsaturated fatty acids. In contrast, samples from the XH site also clustered in the positive range of PC1, with a more dispersed distribution along PC2, indicating higher but more variable unsaturated fatty acid levels (Fig 5B, Table 6). Overall, the PCA results demonstrated that *V. sativa* grown in the MX and XH sites tended to accumulate higher levels of total unsaturated, thereby exhibiting superior nutritional quality compared to those from the QY site.

**Table 5:** Eigenvalues and explained variance of principal components for fatty acid composition in *V. sativa.*

Principal Component	Eigenvalue	Explained Variance (%)	Cumulative Explained Variance (%)
PC1	4.744	59.3	59.3
PC2	2.040	25.5	84.8
PC3	0.688	8.6	93.4
PC4	0.280	3.5	96.9
PC5	0.128	1.6	98.5
PC6	0.080	1.0	99.5
PC7	0.032	0.4	99.9
PC8	0.008	0.1	100.0

**Table 6:** Loading values of fatty acid variables on the first two principal components (PC1 and PC2).

Principal Component	Palmitic Acid	Stearic Acid	Arachidic Acid	Behenic Acid	Palmitoleic Acid	Oleic Acid	Linoleic Acid	Linolenic Acid
PC1	−0.423	−0.301	−0.102	−0.071	−0.372	−0.409	−0.437	−0.470
PC2	0.271	−0.212	0.678	0.669	−0.083	0.052	0.031	0.019

### 3.7. Effects of ecological zone and cultivar on nutritional traits based on two-way ANOVA

Two-way ANOVA revealed that both ecological zone and cultivar had significant effects on all measured traits, including soluble sugar, crude protein, crude fat, saturated fatty acids (SFA), and unsaturated fatty acids (UFA) (Table 7). Significant ecological zone × cultivar interaction was also detected for all traits, indicating that cultivar performance varied across ecological environments. Two-way ANOVA revealed that both ecological zone and cultivar significantly affected all measured traits, including soluble sugar, crude protein, crude fat, saturated fatty acids (SFA), and unsaturated fatty acids (UFA) (Table 7). Significant ecological zone × cultivar interactions were also detected for all traits, indicating that cultivar performance varied across ecological environments. For most nutritional traits, including crude protein, crude fat, SFA, and UFA, ecological zone exhibited higher F-values than cultivar, suggesting a greater contribution of environmental factors to trait variation. However, for soluble sugar content, the cultivar effect was comparable to, and slightly greater than, the ecological zone effect. Overall, both environmental and genetic factors contributed significantly to the nutritional composition of *V. sativa*, and environmental effects were generally more pronounced for most traits.

**Table 7:** Two-way ANOVA results for the effects of ecological zone, cultivar, and their interaction on nutritional traits of *V. sativa.*

Trait	Source	DF	F value	P value
Sugar	Zone	2	617.51	<0.001
	Cultivar	3	648.36	<0.001
	Zone×Cultivar	6	33.85	<0.001
Protein	Zone	2	163.00	<0.001
	Cultivar	3	35.57	<0.001
	Zone×Cultivar	6	51.01	<0.001
Fat	Zone	2	231.62	<0.001
	Cultivar	3	21.16	<0.001
	Zone×Cultivar	6	8.70	<0.001
SFA	Zone	2	79.42	<0.001
	Cultivar	3	16.42	<0.001
	Zone×Cultivar	6	7.48	<0.001
UFA	Zone	2	242.54	<0.001
	Cultivar	3	4.3	0.015
	Zone×Cultivar	6	6.03	<0.001

### 3.8. Comparison of forage nutritional quality across regions and cultivars

**Fig 6:**
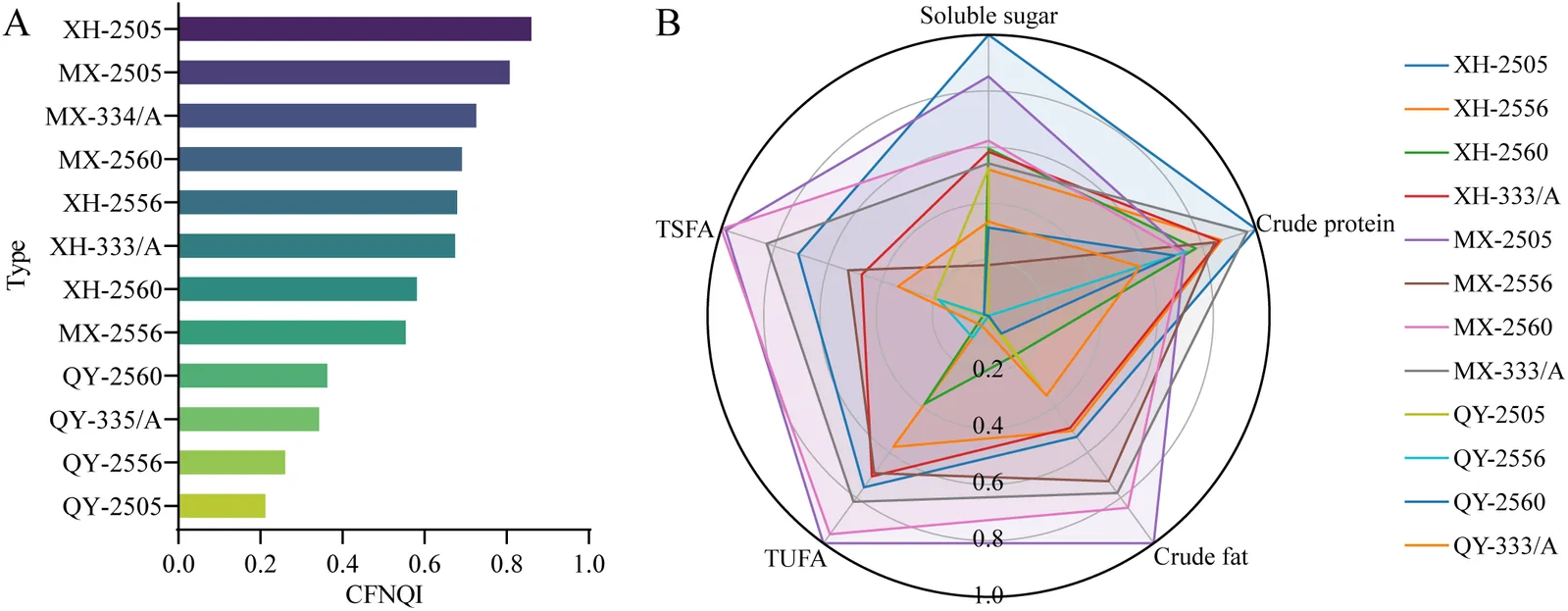
Comparison of forage nutritional quality of *V. sativa.* (A) Nutritional quality scores of different sample types based on the CFNQI results. (B) Scores of samples on five key nutritional indicators, soluble sugar, crude protein, crude fat, total unsaturated fatty acids (TUFA), and total saturated fatty acids (TSFA).

To assess the influence of ecological regions and cultivars on the forage nutritional quality of *V. sativa*, we constructed a new indicator, CFNQI, which integrates five key indicators, soluble sugar, crude protein, crude fat, total unsaturated fatty acids, and total saturated fatty acids. The results showed that CFNQI values for *V. sativa* grown in XH and MX were higher than those from QY, while cultivar-related differences were relatively small. It was demonstrated that ecological factors generally exerted stronger effects than cultivar on most nutritional traits (Fig 6A). Specifically, the XH site, located in the cold alpine climate zone of the Qinghai-Tibetan Plateau, exhibited low temperatures and abundant solar radiation, which facilitated carbohydrate accumulation by promoting soluble sugar synthesis (Fig 6B). The MX site, situated in the transitional zone between the Qinghai-Tibetan and Loess Plateaus, provided moderate temperatures and adequate precipitation, favoring lipid biosynthesis and the accumulation of unsaturated fatty acids (Fig 6B). Therefore, MX is optimal for the production of high-quality fatty acid-enriched forage. In contrast, the QY region, characterized by a temperate semi-arid climate on the Loess Plateau, experienced higher temperatures and lower precipitation, which constrained nutrient accumulation and resulted in relatively poor forage quality, indicating a weaker suitability for high-nutrient forage production (Fig 6B). In summary, *V. sativa* is better adapted for cultivation in cooler, moderately moist environments such as highland or transitional zones, where favorable climatic conditions promote the synthesis and accumulation of nutritionally valuable compounds.

### 3.9. Effects of environmental factors on CFNQI

**Fig 7:**
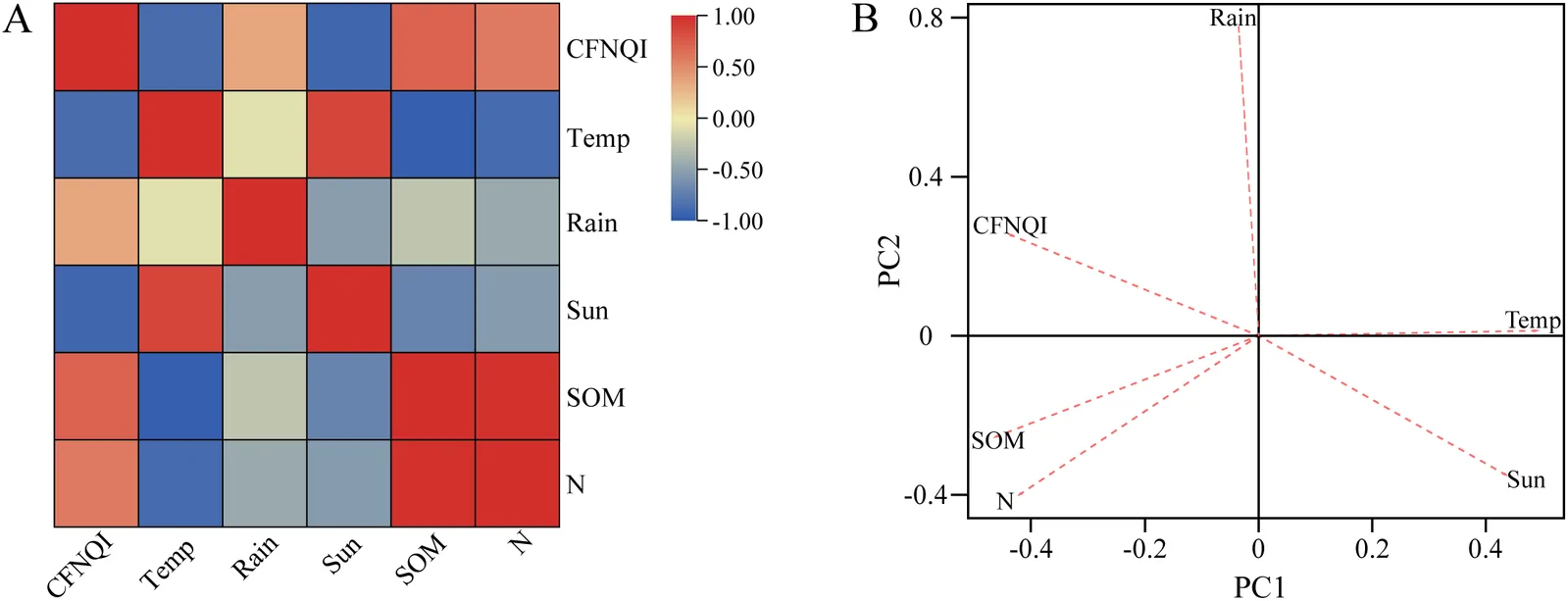
Associations between environmental factors and the comprehensive forage nutritional quality index (CFNQI). (A) Pearson correlation heatmap illustrating the correlation coefficients between CFNQI and key environmental variables (Temp: temperature; Rain: precipitation; Sun: sunshine duration; SOM: soil organic matter; N: soil total nitrogen). (B) PCA biplot showing the ordination of environmental variables and CFNQI along the first two principal components. Red dashed vectors represent the direction and strength of variable contributions.

Pearson correlation analysis revealed CFNQI was strongly negatively correlated with temperature (r = −0.86) and sunshine duration (r = −0.90), and positively correlated with soil organic matter (r = 0.72), soil nitrogen (r = 0.59), and precipitation (r = 0.36) (Fig 7A). SOM and soil N showed strong collinearity (r = 0.98). PCA confirmed CFNQI was associated with high precipitation and soil fertility, but negatively linked to temperature and sunshine duration (Fig 7B). These results indicated that higher CFNQI values were associated with cooler and wetter environments as well as greater soil fertility.

## 4. Discussion

*V. sativa* is a widely utilized leguminous crop with multifunctional value, serving as a source of food, forage, feed, and green manure. It plays an important role in agricultural production systems worldwide, particularly in supplying feed for livestock and poultry [13]. Due to its short growth cycle, high biomass production, strong nitrogen fixation capacity, wide ecological adaptability, high nutritional value, and substantial economic returns, *V. sativa* is well suited for cultivation under diverse climatic conditions. In China’s grassland-based agriculture, it holds an irreplaceable position, especially in high-altitude and cold-climate regions where it contributes significantly to pasture improvement and the development of animal husbandry [12,14]. However, the northwestern regions of China are characterized by harsh ecological conditions and severe degradation of natural grasslands, which has substantially constrained the promotion and utilization of leguminous forage species [37–39]. This study focused on the influence of distinct ecological regions on the growth performance and nutritional quality of *V. sativa*, to evaluate its regional suitability and potential as a high-quality forage resource. The findings provide a scientific basis for optimizing forage crop deployment and promoting the sustainable use of grassland ecosystems.

Multiple factors influence the growth of *V. sativa*, which can be broadly categorized into environmental and genetic factors. Among these, environmental factors are the dominant determinants of the nutritional quality of *V. sativa* [18,40]. The actual accumulation of nutrients is highly dependent on ecological conditions, particularly climatic variables, and soil properties [41,42]. Plants possess physiological regulatory mechanisms that adjust nutrient synthesis and allocation in response to their environment. These regulatory processes mainly involve adjustments in photosynthetic carbon assimilation, respiratory consumption, and metabolic partitioning between carbon and nitrogen pathways. The present study demonstrated that ecological conditions are the primary drivers influencing the nutritional composition of *V. sativa*. The XH site on the Qinghai-Tibetan Plateau has a cold climate, high altitude, and strong sunlight, which help *V. sativa* accumulate more soluble sugars. This may be associated with enhanced light-driven photosynthetic carbon fixation and reduced temperature-induced respiratory losses. The MX site, situated in the transitional zone between the Qinghai-Tibetan and Loess Plateaus, features a moderate climate and balanced precipitation, providing optimal conditions for lipid and unsaturated fatty acid synthesis. Such conditions likely promote carbon flux toward acetyl-CoA metabolism and support temperature-sensitive desaturase activity involved in unsaturated fatty acid biosynthesis. Consequently, *V. sativa* grown in MX exhibited superior lipid nutritional qualities. The arid conditions at the QY site hindered nutrient accumulation, reducing forage quality. Water limitation and higher temperature stress may suppress enzymatic activity and reduce metabolic efficiency, thereby constraining both carbon assimilation and lipid biosynthesis. Similar patterns have been reported in sone studies on legume forage species. For example, research on *V. sativa* and other forage legumes in Mediterranean and temperate regions has shown that environmental factors such as temperature, precipitation, and solar radiation significantly influence biomass accumulation and forage quality, particularly through their effects on carbohydrate and lipid metabolism. In summary, climatic conditions significantly influence the nutrient composition of forage crops by modulating plant carbon-nitrogen metabolism and fatty acid biosynthesis, thereby determining the overall nutritional quality of *V. sativa*.

In addition, investigations on alfalfa (Medicago sativa) and related species have reported that unsaturated fatty acid content is highly sensitive to environmental conditions, especially temperature-dependent regulation of fatty acid desaturase activity. These findings are consistent with our results, indicating that *V. sativa* exhibits similar ecological responsiveness in nutritional trait formation across different geographic regions.

In this study, we observed that the effect of genetic factors on forage nutritional quality was smaller than that of environmental factors, with significant differences among cultivars detected only for certain traits. Previous studies have reported that different germplasms can exhibit significant variation in dry matter yield and crude protein content, and differences in biological nitrogen-fixation capacity may influence crude protein levels in forage [43,44]. Therefore, genetic factors can also play a role in determining forage quality. The results of the two-way ANOVA further supported this pattern, showing consistently higher F-values for ecological zone than for cultivar across most traits, indicating that environmental factors contributed more strongly to trait variation than genetic factors. In addition, significant zone × cultivar interactions suggest that genotype performance was environment-dependent, reflecting the presence of genotype × environment interactions. The relatively small influence of genetic factors observed in our study may be attributed to the large environmental differences among the three experimental planting sites, which exerted a strong effect on forage quality. The magnitude of this environmental influence likely overshadowed the impact of cultivar differences, resulting in the comparatively minor effect of genetic factors on forage nutritional quality. Such environmental effects may regulate plant carbon and nitrogen metabolism, as well as lipid biosynthesis pathways, thereby amplifying differences in nutrient accumulation and reducing the relative contribution of genetic variation.

The CFNQI constructed in this study integrates five key nutritional indicators, providing a more holistic and scientific assessment of forage quality. Among these, soluble sugars contribute to palatability and voluntary feed intake [45]. Crude protein serves as the primary nitrogen source in animal feed, directly influencing growth and milk production in livestock [46,47]. Crude fat, as a high-energy component, enhances the energy density of the diet [48]. UFAs are beneficial for improving meat quality and dairy product composition, while SFAs, though possessing nutritional value, may exert adverse health effects when consumed in excess [27,49]. Compared with single-parameter assessments, the CFNQI offers a comprehensive and practical tool for quantifying overall forage nutritional value, enhancing the accuracy and applicability of nutritional evaluations across different ecological types. The CFNQI results in this study suggest nutritional advantages of *V. sativa* cultivated in the XH and MX regions relative to the QY region, while simultaneously underscoring the limitations in nutrient accumulation under the warm, arid ecological constraints of the QY region. Therefore, CFNQI can serve as an effective quantitative metric for evaluating the ecological suitability of forage crops based on their nutritional profiles.

Relative Feed Value (RFV) and Relative Forage Quality (RFQ) are widely used indices for evaluating forage quality. Both indices are primarily based on fiber-related parameters, such as acid detergent fiber (ADF) and neutral detergent fiber (NDF), and are mainly applied to estimate forage digestibility and intake potential [50,51]. However, these indices do not fully account for other important nutritional components, such as soluble sugars, crude fat, and fatty acid composition. This limitation is particularly relevant for forage species such as *V. sativa*, in which lipid content and fatty acid profiles play an important role in determining nutritional value. In contrast, the CFNQI proposed in this study focuses on a broader range of nutritional components, enabling a more comprehensive evaluation of forage nutritional quality. Nevertheless, CFNQI is not intended to replace RFV or RFQ; rather, these indices are complementary and can be used together to provide a more complete assessment of forage quality.

The results of this study indicate that cool and humid or moderately humid ecological conditions are generally associated with higher forage nutritional quality. Therefore, future forage cultivation and extension efforts should fully leverage the unique advantages of each ecological region. For instance, the XH site is well-suited for the development of high-energy, carbohydrate-rich forage, and the MX site may serve as a key production area for nutritionally superior forage rich in lipids, while in QY, ecological suitability could be improved through optimized irrigation management or mixed-species sowing strategies. Additionally, the two-way ANOVA demonstrated that both ecological zone and cultivar significantly affected nutritional traits, although ecological zone generally explained a larger proportion of variation than cultivar for most traits. Therefore, in the process of forage cultivar development, it is crucial to conduct systematic evaluations of ecological adaptability.

## 5. Conclusions

In this study, a series of field trials of *V. sativa* were conducted across three distinct ecological zones, and a nutritional index, CFNQI, was developed by integrating dry matter and fatty acid traits. The results of two-way ANOVA demonstrated that both ecological zone and cultivar significantly affected forage nutritional traits, although ecological effects were generally stronger than cultivar effects for most traits. Cold and humid environments promoted the accumulation of soluble sugars and un-saturated fatty acids, while moderate conditions favored lipid synthesis. In contrast, nutrient accumulation was constrained under warmer and drier conditions, resulting in lower forage quality. Correlation and principal component analyses further indicated that temperature and radiation were key limiting factors, whereas soil nutrients positively contributed to forage quality. Overall, environmental gradients appear to play a major role in shaping nutrient accumulation patterns in *V. sativa*, and CFNQI provides an effective tool for integrated evaluation. These findings provide a basis for region-specific forage cultivation and quality optimization.
